# Hospital and Institutional News

**Published:** 1914-09-26

**Authors:** 


### HOSPITAL AND INSTITUTIONAL NEWS.

### THE TRANSPORT OF WOUNDED FROM THE FIELD OF BATTLE TO THE BRITISH HOSPITAL.

In the first reports which we gave of the arrival of the wounded in this country there were necessarily a few matters where improvement was called for. All these have now been successfully adjusted, and the existing arrangements reflect the greatest credit upon the Royal Army Medical Corps, the Ambulance Services, and the Military Medical Service throughout. We hope to give in a subsequent issue a fairly complete account of the organisation now perfected. It cannot fail to be of interest to point out that most of the wounded soldiers, belonging to different regiments, who have arrived in London during the current week, many of whom have been provided with accommodation in some of the voluntary hospitals, have been at the Front since the beginning of the war, and the majority had received their wounds eight or ten days ago in the earlier part of the battle of the Aisne River. Some of the wounds were of a serious nature, and the majority were caused by shrapnel, the lesions occurring chiefly in the lower extremities. Hardly any bullet wounds were met with, the shells causing deep flesh wounds and several cases of fracture in the long bones. Some of the more serious wounds were septic. It was very noticeable, moreover, how well most of the wounded looked, and the non-septic character of the majority of the wounds was a remarkable proof of the excellence of the arrangements made by the medical authorities at the Front, and of the care and devotion of those responsible for the patients during their transit through France, across the Channel, by rail, and in ambulances to the hospital. The excellent motor-ambulances which conveyed the patients from the station were supplied by the British Red Cross Society. These facts justify the conclusion that our transport arrangements for the wounded leave little to be desired.

### WELSH WAR HOSPITAL APPOINTMENTS.

At an executive meeting held last week the following were appointed to the staff of the Welsh War Hospital (the scheme for which was described in The Hospital of September 12). The medical officers are Dr. F. Armstrong, formerly resident medical officer at King Edward VII.'s Hospital, Cardiff, and now in practice; Dr. Garfield Evans, M.D., an old Guy's man and now in practice at Port Talbot; Professor Emrys Roberts, M.D., Professor of Pathology and Bacteriology at South Wales University College (who went through the South African War as a dresser); and Dr. J. S. Rowlands, M.D., a Cardiff practitioner, formerly resident medical officer at the Royal Infirmary, Liverpool, and now an anæsthetist at King Edward VII.'s Hospital. The dressers are Messrs. A. A. Prichard, Telford Morgan, Reg. H. Leigh, and Leslie W. Jones. Mr. E. J. August, London, is quartermaster; and the warrant officer is Mr. T. R. Ingledew, Cardiff. The matron is Miss M. Martin, who, formerly a sister at King Edward VII.'s Hospital, was one of those who joined the Welsh Hospital in South Africa. Among the dressers, Mr. Leigh, we may note, is a student at the London Hospital, as is also his colleague, Mr. L. W. Jones. The total staff, with the nurses, when engaged, will amount to forty-eight, and there will be a uniform grant and pay according to rank, with a month's gratuity on completion of service. Contracts for furniture and equipment, drugs, instruments, and dressings have already been signed.

### THE LAST ACT OF RHEIMS CATHEDRAL.

To the destruction of Louvain has now been added the destruction of Rheims Cathedral, perhaps the most famous example of thirteenth-century cathedral architecture in the world. If the accounts published by the foreign correspondents may be trusted, the last preparations before the German departure which the old building witnessed were those for converting it into a Red Cross hospital. If this story is indeed true, it certainly makes the irony of its destruction complete. After describing how the nave and transepts became "a roaring furnace of fire," the *Daily Mail* correspondent continues: "Blazing pieces of carved woodwork crashed down on to the floor of the cathedral where the Germans during their occupation of the town had accumulated great piles of straw intending to convert the place into a hospital. Instantly this caught alight." The writer went on to describe how about twenty of the German wounded who had been carried into the cathedral to justify the use of the Red Cross flag, would have been burnt alive by their own countrymen had not the French Army doctors at their own risk caused them one by one to be carried out by one of the side doors. Only the courage of the priests, we are told, prevented vengeance being taken on the German wounded. It would not have been easy to conceive how the last act centring round the cathedral of Rheims could have been most worthy of the spirit of faith which raised it in the thirteenth century, but this respect for the wounded enemy and repudiation of revenge by its priests will surely live as long as the memory of its Gothic architecture. Even the Red Cross did not save it at last.

### UNQUALIFIED ASSISTANTS FOR INFIRMARY STAFFS.

The great trouble Poor-Law authorities are experiencing in their efforts to maintain an adequate medical staff in their infirmaries is exemplified in the case of the Lambeth Board of Guardians. At the outbreak of hostilities Dr. G. F. Stebbing, assistant medical officer, joined the *Leviathan*, and now Dr. Watkin, third assistant medical officer, and Dr. Reader, sixth assistant medical officer, have accepted commissions for the Army and are awaiting orders. As soon as Dr. Baly, the medical superintendent, heard that Dr. Watkin intended to leave, he advertised for another assistant medical officer, but he informed the Guardians that he did not anticipate being able to fill these posts in a satisfactory manner. Under the circumstances he recommended that Dr. Miller and Dr. Keats be promoted to the posts of third and fourth assistant medical officers respectively as the vacancies occurred, and that clinical assistants—*i.e.* unqualified men, who would shortly be entering for their final examinations, be employed. He had made inquiries and found that unqualified men were being offered high fees to act as assistants to private practitioners, but considering the exceptional experience that could be obtained in an infirmary, he believed it would be possible to obtain the services of the best type of men for their board and residence, and an honorarium of fifteen guineas per quarter. They would relieve the permanent staff of a considerable amount of the less responsible duties, and financially this arrangement would be economical. The fact that there will only be three instead of six qualified assistants during the coming winter will considerably add to the responsibilities of the medical staff, and if there is any considerable increase in distress the outdoor work will become onerous. As the number of admissions to the infirmary is to some extent controlled by the district medical officers, Dr. Baly would like to undertake as much of this work as possible, providing he could do so without seriously interfering with his other administrative duties. The Guardians feel they had no option except to adopt the proposal of the medical superintendent to engage clinical assistants.

### ILLNESS OF MR. HOWARD COLLINS.

All who know Mr. Howard J. Collins, whether personally or by the great reputation which he has made for himself as house governor of the General Hospital, Birmingham, will learn with deep regret of his serious illness. A fortnight ago he was compelled to undergo a serious operation, and at the time of writing his condition is still described as critical. Mr. Collins' illness at the present time is doubly regrettable, on public as well as private grounds, since the influx of the wounded into Birmingham has already been considerable, and such a new extension of work, and opportunity for again showing the patriotic readiness of the voluntary hospitals in the emergencies created by war or legislation, would have been thoroughly congenial to his keen enterprise and administrative ability.

### HOSPITAL APPEALS IN WAR-TIME.

How much this subject is exercising the minds of hospital managers may be judged by the letter from a provincial secretary which we publish this week, as the first outcome of the suggestion from a Glasgow secretary to which we alluded in the previous issue. Pending further correspondence on the subject, which we cordially invite, attention may be drawn to the expedient resorted to at Cardiff. Here Mr. Leonard D. Rea, the secretary-superintendent, has issued a circular-letter with the delayed publication of the annual report, in which he draws attention to the fact that while the 100 fully equipped beds set apart for the wounded help King Edward VII.'s Hospital "to fulfil its Imperial obligation," no neglect of its primary duty to the civil population is occurring. He adds to the hope that subscribers will maintain their usual subscriptions, that there is a danger of overlapping, but that there "lies at the doors" of the charitable of Cardiff "a recognised and thoroughly efficient organisation" for wounded soldiers and civil population alike, on which support cannot be wasted. Since the issue of this letter one gift of £50 has been received, which we trust is only the first indication of public recognition of additional need for support which our voluntary hospitals in Cardiff and elsewhere require at the present time. Wounded began to arrive at King Edward VII.'s Hospital on Sunday, September 13.

### HOSPITAL FINANCE IN TIME OF WAR.

The offer to place fifty beds at the disposal of the military authorities which has been made by the committee of York County Hospital has been accepted, but Mr. W. F. H. Thomson, chairman of the house committee, has assured the governors that if and when this offer is acted upon the needs of the civil population will not be passed over. At a recent meeting, in the course of a speech, he stated that the hospital had already experienced the effect of the war by the diversion of subscriptions, and that the sub-committee which had been appointed to secure increased subscriptions and attract fresh support had had their work interrupted by the war before its completion. At the same time the number of in-patients received since the beginning of the year has increased by 100, and the number of out-patients for the same period has also increased. This throws a significant light on the working of the Insurance Act, and has the effect of making the hospital require funds to meet the additional expense of increased work at the very time when charity is being diverted practically into one channel. This raises a very important point as to the financial policy which the voluntary hospitals should pursue in time of war. We hope to return to this subject at an early date, and in the meantime would be very glad to receive our readers' opinions on the matter. A letter on the subject appears on page 706.

### A NEW CORNISH HOSPITAL OPENED.

Owing to the efforts made by the Daughters of the Cross at the Downes, the long-projected hospital at Hayle, Cornwall, was opened a few days ago by the Rev. Prior McElory, of Bodmin Priory, under the name of St. Michael's Hospital. The building, which is of Cornish granite, consists of a ground floor and two storeys, and provides accommodation for twenty patients in two wards of ten beds apiece, together with a private ward and an operating room, a medical consulting room, an observation room, etc. The two wards measure 40 ft. by 20 ft. each. In opening the institution the Rev. Prior reminded those present that the Sisters were trained nurses, whose work was peculiarly the relief of suffering irrespective of creed, and that the public were to be congratulated on the special advantage, which was possessed by no other town in Cornwall, of having a hospital started and staffed by trained nurses. The hospital is being offered to the War Office for the use of the wounded, and he relied on the generosity of the neighbourhood to help towards furnishing the building, which would become at once necessary if the offer were accepted by the War Office.

### DONORS IN TOUCH WITH HOSPITAL BOARDS.

The recent monthly meeting of the Board of Governors of the Leicester Royal Infirmary was rendered additionally interesting by the fact that Mr. Joseph I. Hallam, one of the institution's life governors, attended and formally asked the board's acceptance of the series of seven electrical clocks and masterpiece which he had installed in the new Children's Hospital. He said that he had assured himself by personal inquiry that the gift was of much service to the nurses and patients, and so had abandoned the idea of handing over the cost in cash. Mr. L. T. Topham, High Sheriff, who was in the chair, voiced the feelings of gratitude of the board for this and many previous instances of Mr. Hallam's interest, in which he was supported by the Chairman of the house committee, Mr. T. Fielding Johnson, junr., and Mr. G. Tempest Wade. We are very much pleased to see willing donors brought in this way into touch with the management of the Leicester Royal Infirmary, and the incident might be multiplied to the advantage of many other institutions, as even in these serious times friends are never lacking to our voluntary hospitals when once their real needs are made known.

### NEW HONORARY SECRETARY OF BLACKPOOL HOSPITAL.

The final touch to the settlement of the dispute between the Blackpool Victoria Hospital's board of management and the local medical men, reported at the time in our columns, was given at the recent adjourned meeting of subscribers. Alderman Mather presided, and a statement was made by Mr. W. J. Read, who announced that with the appointment of Dr. Butcher as hon. secretary and Mr. Spedding as hon. treasurer two vacancies had been created on the board. To the satisfaction of the meeting he declared that the board had thereupon requested Dr. Dunderdale and Dr. McIntosh to accept the two seats. A motion formally requesting the medical men's acceptance was carried on the motion of Councillor Wilde, one of the late mediatory committee. He and Councillor Collins agreed that under the settlement four doctors had to be elected on the board, though another member was of opinion that their election was not necessary until the trust-deeds were reconstructed. If the chairman's concluding hope is fulfilled, that as many of the board had done their best for the hospital they would not bear any animosity towards it, there should be no excuse for further interference with the hospital's work. Its prestige, and therefore usefulness, have inevitably suffered much, but they can be built up again by hard work and the hearty co-operation of all sections of the staff, who will now, we trust, make this their immediate and earnest endeavour.

### UNIQUE GIFT TO THE SEAMEN S HOSPITAL.

An unusual and very interesting gift has been made to the Seamen's Hospital Society by Mrs. E. Spencer Stidolph. It consists of a freehold property in East Greenwich, known as Highbridge House, situated in one of the most picturesque portions of the foreshore of the Thames. The building, which was probably erected over 200 years ago, is of wood, and until about twenty years ago was a portion of the Crown and Sceptre Inn, one of the most ancient hostelries of the Metropolis. The present structure rises from the river's bank and extends along the foreshore for nearly 100 feet. At high water the Thames flows against the walls of the buildings. On the ground floor is a large room with entire glass front, facing the river, and at the west end of it an old boathouse has been covered in and made into a sort of recreation room. On the floor above there is one large room, formerly the ballroom of the hotel. The whole affords a suitable site for the erection of modern buildings. At the rear of the building is an open space, upon which there is an ancient cottage and barn, a part of this latter having been converted into a rifle range. The adjoining premises, which are not included in the gift, are occupied by the Curlew Rowing Club—in former times this was the portion of the hotel devoted to sleeping accommodation. During the last few years the premises have been occupied by the East Greenwich Conservative Club, in which the late husband of the donor took an interest. In this ancient inn were served in olden days the famous Greenwich whitebait dinners, and it was here that Charles Dickens frequently gathered with his many friends. The "Greville Memoirs" mention this ancient tavern, and among the illustrious who have partaken of its hospitality are the Duke of Wellington and Talleyrand.

### NURSES' HOME OR WARD FOR WOUNDED?

It is not yet certain to what use this property will be put, but it has been suggested that it may prove a solution to the difficult question of housing the nursing staff of the *Dreadnought* Hospital. At the present time the nurses sleep two or more in a single room, and although the accommodation is airy and admirable in many respects, the space afforded does not meet the requirements of a hospital of 250 beds. Should a sudden influx of wounded be received at Greenwich after a naval engagement the spacious premises by the river would be immediately available, and would form excellent wards for sick and injured naval ratings. It should be added that the gift above recorded is not the first benefaction Mrs. Stidolph has made to the Seamen's Hospital Society. Both she and her late husband are old benefactors of the hospital, and this gift is further evidence of her desire to aid the Seamen's Hospital in its work for the men of the sea.

### LAY HOSTILITY TO HOSPITAL EFFICIENCY.

At a recent meeting of the Borough of Hornsey Tradesmen's Association, presided over by Mr. Walter Brown, a letter was read from Mr. A. B. Peters protesting against the decision of the Council of Hornsey Cottage Hospital to erect a mortuary and *post-mortem* room, which he characterised as "an unnecessary and extravagant enterprise." While asking the Association not to withdraw their support from the hospital on this account, Mr. Peters urged that they should stipulate that no part of their contributions should be devoted to this extension. Ignorance could not well go further. In The Hospital of March 16, 1912 (p. 597), we reported a speech by Sir Henry Burdett on the subject of cottage hospital mortuaries, which he delivered in opening a bazaar in aid of Tonbridge Cottage Hospital. "I attach," he said, "great importance to the mortuaries of cottage hospitals. For a cottage hospital should set an example in hygiene to the whole community which it serves, and it should also set an example of reverence for the dead. It is extraordinary to me what an absence of this reverence one sometimes meets with in public institutions . . . and therefore I hope the people of Tonbridge will not be content to have any apology for a mortuary—the sort of coalhole with a top that is sometimes found—but that you will have a proper mortuary with a mortuary chapel, where friends may see their dear departed. You should also have an additional room for medical purposes."

### THE VALUE OF COTTAGE HOSPITAL MORTUARIES.

In commenting on this speech we said that the answer to those who, like Mr. Peters, objected to so high a standard being set in these days when subscriptions are hard to come by, is simply that a similar standard is universally insisted upon in every other department of hospital work. We therefore hope that the Council of the Hornsey Cottage Hospital, far from being deterred by what is really an ignorant point of view, will not only press on with the work, but will do their best to bring home to the local Tradesmen's Association the true facts indicated above, in which case we have little doubt that Mr. Peters and his associates will gladly make the *amende honorable*, and admit, by an enhanced subscription, that the Hospital Council were right and themselves wrong in thinking that a hospital's duty to patients and their friends is done when discharge or death has taken place.

### PROFESSIONAL PROSPECTS OF MEDICAL VOLUNTEERS.

In view of the complaints which have been made on behalf of certain medical practitioners, who have volunteered their services to the War Office and been put off, as the phrase goes, with the statement that they will be accepted if called upon, a statement made by Mr. Tennant, Under Secretary of State for War, in the House of Commons last week should make the position clear. He was asked by Mr. Stewart what were the prospects as to getting appointments for medical men who had volunteered for service and had been accepted and medically examined by the War Office. There were cases, he proceeded, where medical men on acceptance had resigned all professional work and found themselves without position or income pending the final acceptance of their services. Mr. Tennant, in reply, said that of some 900 who applied (and it is interesting to have this figure), about 70 per cent. had been employed. It was, he added, made clear to the remainder that their acceptance for service, should they be required, did not place them under any obligation to relinquish their ordinary appointments until actually called upon to serve. In effect this means that the complainants referred to have only themselves to blame, and our sympathy with their eagerness must not blind us to the fact that the War Office's attitude seems to have given no real ground for misapprehension.

### THE TRUTH ABOUT HOSPITAL TRAINS.

In view of the rapid transportation of wounded to England the hospital ship has received more public attention than the hospital train, but those who place side by side the two following accounts, which appeared in the *Times* of Monday last, from its own correspondents—at La Ferté Milon, a layman; and at somewhere near Orleans, a "medical" writer—will find an instructive contrast. Describing the struggle on the Aisne the lay correspondent said: "I have seen the wounded pass, and there is no end to them. Every evening the trains come crawling past . . . silent passenger coaches with the great Red Cross painted on their sides, thirty coaches to a train. . . . Each carriage is crammed—packed fuller with wounded than any excursion train with holiday-makers in time of peace. The worst cases are lucky if they can lie at full length. . . . In this war of to-day," concludes the correspondent, "when the battlefield is covered day after day with thousands and tens of thousands, there is no use for comfortable hospital trains which can hold a couple of hundred at most. I have seen these trains of wounded; I have travelled in them day and night . . . and there was not room for one more. In that single train there must have been at the lowest estimate a thousand men." In this statement the hospital train is condemned as hopelessly inadequate. Writing the same day from the lines of communication near Orleans on the precautions taken against infection, the "medical" correspondent states: "Visit a railway station when a train of wounded comes down from the front. Note the quality of the dressings—how clean they are, what trouble has been taken to prevent infection—the deadly enemy of military hospitals. Note also that the wagon in which they travel—for the wise principle of swinging the stretchers in roomy wagons has been adopted—bears a label 'Infected.'" Presumably the "they" who travel thus comfortably refers only to "infected" cases, for whom crowding together like holiday excursionists would be more than disastrous; otherwise the accounts are difficult to reconcile. It is to be observed, however, that the comfortable picture comes from lines of communication south of Paris, and relatively far from the fighting line; the lay writer is on the heels of the battle. May not the truth be that the number of hospital trains available for the Front are too few for the dreadful demands of modern warfare?

### THE HOSPITAL CLASS AND THE FOOD QUESTION.

Now that the war has brought home to us the possibility anyway of high prices, especially in the case of food, some medical officers of health have drawn up leaflets showing how as much nourishment can be obtained by normally inexpensive foods as by those which, like meat, have become to an enormous degree the staple of poor housekeepers. In pointing out without technical language that carbohydrates, proteids, and fat are necessary constituents of a nourishing diet, and that such vegetable foods as dried peas, haricot beans, and lentils roughly contain equivalent proteid to meat, while being much cheaper, the medical officers hope to pave the way to a more economical working-class housekeeping. We cannot shut our eyes to the fact, however, that nothing short of necessity will make the housewife, or her husband, forgo their staple articles of diet, whatever proof may be forthcoming of cheap and efficient substitutes. The real reason of this we believe to be not so much tenacity of habit as the deep-rooted conviction that reduced cost of living means also a fall in wages. That is why economy in his food will seem to him always a foretaste of reduced wages, and, as scarcity and high prices of food caused by war are accompanied by unemployment, there is something more than superstition in his point of view. Whether well-informed or ill-informed, however, medical officers of health had better realise what one of their main difficulties will be in their endeavour to drive home the value and possibility of a much more economical diet.

### THE STANDING GRIEVANCE OF SOUTHWARK INFIRMARY.

Over twelve months have elapsed since Dr. Bruce, the medical superintendent at the Southwark Infirmary, drew attention to the nuisance arising from a dust-siding, close to the infirmary, used by the Camberwell Borough Council. A serious outbreak of enteritis occurred amongst the children, when nine died, and Dr. Bruce attributed the outbreak to the nuisance from the dust-siding. How the Guardians laid the facts before the Local Government Board, who called on the Camberwell Borough Council to discontinue the use of the siding after it had been visited by Dr. Fletcher, an inspector specially sent down from Whitehall to investigate, is an old story. So is also the refusal of the Camberwell Borough Council to do more than use a screen to prevent, if possible, the dust from blowing into the infirmary wards. This summer, as is not surprising, saw a recurrence of the nuisance, when again the Southwark Board of Guardians appealed to the Local Government Board, who at last consented to receive a deputation. The deputation was informed that the Local Government Board had no power to compel the Camberwell Borough Council to abate the nuisance, but after twelve months' delay the Guardians must go to the London County Council, who, to their great surprise they were told, might eventually refer the matter back to the Local Government Board. It would appear that the Local Government Board were afraid that there was not sufficient legal proof that the nuisance existed so acutely as to be a danger to public health, though they might be convinced in their own minds that it was prejudicial to the health of the inmates of the infirmary.

### THE IMPOTENCE OF THE LOCAL GOVERNMENT BOARD.

The above decision was strongly criticised by the Southwark Board of Guardians, and the deputation protested. They were informed that there were grounds for taking action against the Camberwell Borough Council under the Public Health Acts, and had it been a private individual and not a public authority that committed the nuisance complained of they would speedily have been summoned to abate it in a court of summary jurisdiction. Here, however, there are two public authorities, and whether the Guardians could take action by way of summons in a police court against the Camberwell Borough Council, who are responsible for the health of the borough, for committing a nuisance within their own borders is a matter that the legal authorities would have to decide. The Guardians are determined not to let the matter rest, but have decided to appeal to the London County Council to help them in endeavouring to make Camberwell Borough Council do its duty as a public health authority. Whether they will be more successful with the London County Council than they have been with the Local Government Board remains to be seen. If they are not, then it rests with them, in the interests of the patients in their infirmary, to see if the law's delay is as great as that of local authorities and public departments, for the attitude of the Local Government Board is nothing but a confession of impotence.

### DR. MACKINNON MEMORIAL HOSPITAL OPENED.

To the relatively few institutions for the sick dedicated to the memory of medical practitioners must now be added the Dr. Mackinnon Memorial Hospital, Broadford, N.B. The initiative was taken by Lady Macdonald and the Rev. Mr. Cameron, Sleat, to commemorate the late minister of Strath, who, in the words of the hon. secretary, Mr. James Campbell, "by his skill in medicine, which he studied at college, was equal to the care of the bodies as well as the souls of his parishioners." The architect was the late Mr. Carruthers, of Inverness.

### HOSPITAL WORK IN DUNDEE.

Some interesting sidelights were thrown on the effect of the war upon normal hospital procedure at the quarterly meeting last week of Dundee Royal Infirmary. The number of patients, Mr. James Guthrie explained, had been, of course, considerably reduced. Seven of the medical and surgical staff and ten nurses have left for duty in naval or military hospitals. Moreover, owing to large numbers of doctors on the Insurance list having been called up for service with their Army units, whose colleagues have agreed to undertake their work, the directors of the Royal Infirmary have placed rooms in the institution and in the out-patient department at the disposal of the local medical committee for carrying out the work of registration of cases. This is only the latest example of the way in which the voluntary hospitals have come to the rescue over the problems of institutional treatment and medical practice raised by the Insurance Act. But it is none the less worth noting on that account. Lieutenant-Commandant Still, the chairman, has left in command of the Royal Naval Volunteer Reserve. So from every point of view we find, as usual, the voluntary hospital being the reserve of assistance to the emergency, whether created by home legislation or European war. As to the children's hospital scheme, the directors have reported that as it would cost £40,000, apart from a site, for 100 beds, an endowment of £100,000 should be raised before proceeding.

### THE AUSTRALIAN AMBULANCE FORCE.

One after another news of the organised efforts of the Dominions to despatch Red Cross units, whether as ambulance organisations or hospital ships, comes to hand. The first expeditionary force despatched from Australia was accompanied by its own ambulance organisation, but in addition a special Australian ambulance force, says the *Morning Post* correspondent, is to be sent out. It is to comprise a light horse-drawn field ambulance, a clearing hospital, a field ambulance, two stationary and two general hospitals, with a total accommodation for two thousand patients. The ambulance force is being accompanied by eighty medical men and by a staff, of all ranks, amounting to one thousand. Moreover, the Red Cross Society has forwarded £15,000 to London. These brief facts suffice to show the scale on which the ambulance force is being organised, and the generous spirit which has prompted it. All that we can say at present is that the first phase of the war, which may probably rightly be regarded as now over, has shown that the casualties are likely to be no less heavy than was at first feared; and therefore that every properly equipped and trained ambulance is likely to be quite as welcome as are the contingents of troops so readily offered by the Dominions.

### SECRETARY OF WORCESTER INFIRMARY RETIRES.

At the monthly meeting last Monday Canon Longhurst referred to the approaching retirement of Colonel Stallard from the secretaryship, and expressed his own feeling of thanks for the excellent work he had done for Worcester Infirmary. The Chairman said that he had always received the greatest courtesy and help from Colonel Stallard, and he hoped that, although he would cease to be secretary, he would remain a member of the board. In replying, Colonel Stallard said that his thirty years in the service of the infirmary would always be remembered by him with pleasure. Will the committee now carry out the advice that we have so often ventured to tender? For instance, in The Hospital of August 16, 1913, in a leading article, we strongly urged that instead of reducing the secretary's salary by 20 per cent. "the services of a trained and competent hospital administrator as secretary-superintendent, at an adequate salary, should be secured, who will devote his whole time and energies to the work." A committee of business men backing such an official we confidently predicted would make the infirmary's financial position sound in six months.

### THE CHURCH ARMY WAR HOSPITAL.

The Church Army has organised and equipped an Army War Hospital, which has been sent out to act in conjunction with the French Red Cross Society, and is now at work in North France. The staff consists of Doctors Alfred C. T. Woodward, Francis Graham Crookshank, and Allan Pimm, and there are two dressers, a dispenser, six trained nurses, and fourteen orderlies. The latter are Church Army evangelists holding the certificate of the St. John Ambulance Association. The hon. adviser is Sir Rickman Godlee, and Lady Bagot, R.R.C., is acting as hon. secretary.

### THE WEEK'S DRUG MARKET.

Business continues very quiet, but there is a steadier tone about the market, and fluctuations in prices are less general and less violent. The demand for drugs which had become very scarce has to some extent and in some cases been met by recent arrivals from foreign ports; the United States, Holland, Belgium, Italy, Portugal, and Norway are all contributing to our stocks, but naturally the supplies received from these sources are restricted in quantity. In America there is a scarcity of German-made drugs, and consequently little can be spared from that country for exportation to ours. A drug which has advanced to a very serious extent and is extremely scarce is belladonna root; very high prices are being paid for the small quantities available. Quinine is practically unchanged in price; this is very unusual at times of war, but there are large stocks in the country at the present time. Opium is another drug which has not advanced in price to anything like the extent that might have been expected; the reason is that supplies are comparatively plentiful.

